# Flowers in the Sick Room

**Published:** 1884-04

**Authors:** 


### FLOWERS IN THE SICK ROOM.

THE "Superstition," as he called it, that plants are not healthful in sleeping or sick rooms, was vigorously attacked by Dr. S. M. Andrews recently in a lecture before the sociel meeting of the alumni of the College of Pharmacy, Philadelphia. The deleterious matter that they gave out, the doctor declared, is too small to have any appreciable effect, while their positive value in the sick room is great. They fulfil two functions—that of the generation of ozone and exhalation of vapor, by which the atmosphere of the room is kept in a healthful condition of humidity. Tests made by the doctor at Christ Hospital, showed that, in two rooms, alike in all respects, except that one contained some flowers and the other none, that containing the flowers was cooler, by 1½ degrees than the other room.

The ozone which is generated by budding and flowering plants the doctor had found to have great sanitary value, in that it purified the air, ridding it of disease-breeding germs and of the vapors of decomposition. For consumption, ozone is ofgreat benefit, arresting the course of the malady; and by living among flowers constantly, consumptives have been known to reach an advanced age. Of thirty florists whom the doctor visited, he found none who had consumption, though among the families of several it was hereditary. Foliage plants, the doctor found produced no ozone, and, as far as he had experimented, he had found no difference between odoriferous and non-odoriferous plants. More experiments were urgently advocated to determine more definitely the value of this new remedy for consumption.

